# A Structural Equation Modelling Approach to Examine the Relationship between Socioeconomic Status, Diet Quality and Dyslipidaemia in South African Children and Adolescents, 6–18 Years

**DOI:** 10.3390/ijerph182312825

**Published:** 2021-12-05

**Authors:** Gugulethu Moyo, Esteban Montenegro-Montenegro, Zachary Stickley, Abdulkadir Egal, Wilna Oldewage-Theron

**Affiliations:** 1 Department of Nutritional Sciences, College of Human Sciences, Texas Tech University, 1301 Akron Ave, Lubbock, TX 79409, USA; Wilna.Oldewage@ttu.edu; 2 Alzheimer’s Disease Center East-Bay, University of California, Davis, CA 94598, USA; emmontenegro@ucdavis.edu; 3 Institute for Measurement, Methodology, Analysis & Policy, Texas Tech University, Lubbock, TX 79409, USA; zachary.stickley@ttu.edu; 4 Centre of Food and Nutrition Security, Somali National University, Mogadishu, Somalia; Egal_a@yahoo.ca; 5 Department of Sustainable Food Systems and Development, University of the Free State, Bloemfontein 9300, South Africa

**Keywords:** non-communicable, health, disparities, diet quality, socioeconomic, children

## Abstract

This study utilised a structural equation model to examine the relationship between diet quality, socioeconomic status, and cardiovascular disease (CVD) risk in South African learners. Confirmatory factor analysis was used to test the indirect effects model for diet, socioeconomic status, diet quality and cardiovascular risk using pre-existing cross-sectional data. The structural equation model was fit using Lavaan version 0.6–5 in R version 3.6.1. Data were analysed from 178 children and adolescents, aged 6–18 years, from five rural schools in Cofimvaba, South Africa. Latent variables were created for dietary quality, dyslipidaemia and the socioeconomic status of participants. A negative association was observed between socioeconomic status and dyslipidaemia in school-aged children (*p* = 0.029).

## 1. Introduction

While infectious diseases have reduced significantly over the past decades, non-communicable diseases (NCDs) have been on the rise [1,2]. Cardiovascular disease (CVD) is the leading global cause of death, responsible for approximately 18 million deaths per year in 2016 and projected to increase to over 23 million by 2030 [3]. South Africa (SA), like most developing countries, is faced with the “double burden of malnutrition”, a phenomenon where under- and over-nutrition occur in the same population and sometimes the same individual [4,5]. This can be explained in part by the nutrition transition, a shift in dietary behaviours towards a more Western diet, which is high in processed foods, and a reduction in physical activity [2,6].

CVD is responsible for almost one in six deaths (17.3%) in SA, which is more than all cancers combined [4]. Studies have found that diet and socioeconomic status contribute to CVD risk [7,8,9,10] and that CVD risk factors can be present even in childhood [11,12]. Lower socioeconomic status (SES) has been associated with various dietary patterns such as low fruit and vegetable intake [13,14,15,16,17] and high added sugar consumption [18,19,20]. In 2011, the South African Summit on the Prevention and Control of NCDs highlighted the importance of strategies that include early identification and behaviour modification [21].

Studies from the United States (US) and Europe have shown a relationship between the presence of dyslipidaemias in childhood, increased risk of overweight in late adolescence [11], increased carotid intima-media thickness, a marker of subclinical atherosclerosis, in later life [22] and future coronary heart disease [23]. Dyslipidaemia, a risk factor for CVD, is a condition that is characterised by an abnormal amount of lipids in the blood, for example, triglycerides, total cholesterol, high-density lipoprotein (HDL) cholesterol and low-density lipoprotein (LDL) cholesterol [24]. Since CVD risk begins in childhood [25] and with the growing prevalence of NCDs in SA, a study was conducted to address the paucity of data available on the lipid profiles of South African children and adolescents [12]. A disturbing finding from this study was a high prevalence of low HDL cholesterol among children aged six to nineteen, indicating a high risk of developing CVD [12]. Furthermore, high-sensitive C-reactive protein (HS-CRP) levels were observed in one out of five children, indicating inflammation [12].

There are no known studies that sought to understand the pathways leading to increased dyslipidaemia in South African children from poor households. For SA to develop appropriate strategies to address the high prevalence of dyslipidaemia observed in children, it is important for the country to identify which issues to target when designing interventions. While diet is the main source of fat, studies do suggest that fat intake is not the only issue and that lifestyle and economic factors must also be taken into consideration [7,8,9,10]. Structural equation modelling (SEM) is a technique that is underutilised in the field of nutrition research, despite calls for its use in nutritional epidemiology as an integrated, theory-guided approach to examining the complex relationships that exist between determinants of nutrition, health and related behaviours [26]. This study aimed to utilise SEM to examine the relationships between diet quality, socioeconomic status and dyslipidaemia in South African children.

## 2. Materials and Methods

### 2.1. Study Design and Participants

Secondary data analysis was conducted on a cross-sectional data set of school-aged children (6–18 years old) in the Intsika Yethu municipality, Eastern Cape.

An adequately powered sample for the original study that this data were collected for was 235 participants. This was based on a sample size calculation that considered a 95% confidence level, a 6·25 CI and the total number of public school children (*n* = 5250) in the study area. Permission to undertake the study was obtained from five schools and these were thus purposively selected based on them catering to mainly Xhosa speaking, low-income households. Cluster sampling was then conducted based on gender, age and school, and 240 participants, from whom parental consent was obtained, were selected. Data were obtained for 237 children. Ethical approval for this study was obtained from The Senate Research and Innovation Ethics Committee of the Vaal University of Technology (20130520-3). Further details on the sampling methodology and ethical clearance have been published previously [12].

We confined our analysis to information on the dietary intakes, blood lipid profiles, and parental SES. For the purposes of the current analysis, the school variable was ignored, and all children were treated as one group.

### 2.2. Study Data Collection Tools

#### 2.2.1. Dietary Assessment

Food frequency questionnaires (FFQs) were used to collect information on a wide range of foods consumed in the previous 24 h, for example, fruit and vegetables, meat, eggs, milk, salt, and sugar. Dietary diversity scores (DDS) and food group diversity scores (FGDS) for fruit and vegetables were calculated from the FFQ. Twenty-four hour recall data were collected with the assistance of trained fieldworkers using food models to estimate portion sizes for children aged 10 and older. The same procedures were used for the younger children in one-on-one interviews with the assistance of the parents/legal guardians [12]. To improve the validity and reliability of the data, a four-stage, multiple-pass interviewing procedure was used for the 24 h recall questionnaires, and they were administered on one weekday and on one day of the weekend one week apart. The 24 h recalls were analysed using the Food Finder software program (version 3, Cape Town, South Africa) to provide the amounts of various nutrients consumed [12].

#### 2.2.2. Biochemical Measurements

A registered nurse collected blood samples from the children. Blood was drawn using a vacutainer needle before 10 am following an overnight fast, and breakfast was served immediately afterwards [12]. Low-speed centrifugation at 4 °C was used to separate the serum that was then aliquoted into tubes and stored at −80 °C. Lipid analysis was carried out for total cholesterol, HDL-C and triglycerides on the (Thermo Fisher Scientific, Holliston, MA, USA) analyser (colorimetric method). LDL-C was calculated using the Friedewald method [12].

#### 2.2.3. Assessment of SES

Household SES was collected using a pre-tested and validated socio-demographic questionnaire administered to the parent or primary caregiver. All questionnaires were administered by trained enumerators and included multiple-choice questions about household size and composition, education level and employment status, monthly household income, marital status, asset ownership and living conditions (electricity, potable water and sanitation). In addition, the age and gender of the caregiver and child were recorded [12].

### 2.3. Development of Latent Variables

There is currently no consensus on the best variables to use to create a comprehensive SES latent variable. However, due to the results of the correlation matrix and support from existing literature [27,28,29], income and employment were used as proxy indicators.

Prior research suggests that dietary quality, specifically the intakes of fresh vegetables and fruit, whole grains and fish, has been found to have a protective effect on CVD, whereas a high intake of added sugar, sodium and refined carbohydrates [7,30] and dietary fat, particularly trans fatty acids, have been identified as a risk factor for CVD [31,32]. Dyslipidaemia is also a risk factor for CVD and, since the 2000s, the prevalence of dyslipidaemia among children has been on the rise [33]. Existing literature was used as the starting point for the selection of indicators for each latent variable. This was later refined by creating a correlation matrix to identify correlated variables.

The covariance matrix revealed that in this population, total serum cholesterol and low-density lipoprotein cholesterol were strongly correlated; therefore, these two were used as indicators for dyslipidaemia. Initially, we attempted utilise the commonly known CVD risk indicators to create the latent variable for dyslipidaemia (e.g., triglycerides, LDL-cholesterol, HDL-cholesterol and HS-CRP). However, we found that only cholesterol indicators had statistically significant loadings in the structural equation model; therefore, the other indicators were excluded from the final diagram.

To measure the diet quality latent factor, fruit intake, vegetable intake, dietary diversity score (calculated by summing the number of unique food groups consumed over the last 24 h) [34], added sugar and total fat intake were used. These indicators were also selected based on a preliminary analysis conducted using a covariance matrix.

### 2.4. Data Analysis

**Figure 1:**
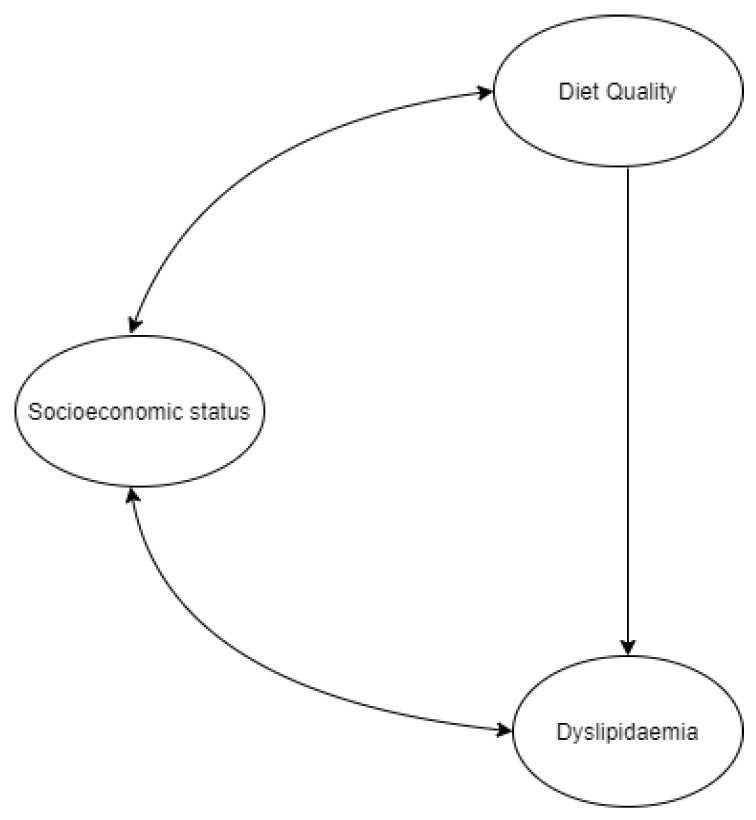
Hypothesised model.

Descriptive statistics (means ± SDs) were calculated in R statistical package version 3.6.1 [35] for observed variables and compared to the reference values for each parameter. A conceptual framework was developed to illustrate the possible interactions among the latent variables (Figure 1). To analyse the process by which diet quality and socioeconomic status were related to dyslipidaemia in children, SEM was performed on the total sample, based on the model hypothesised in Figure 1. The double-headed arrows in the figure represent correlations, while the single-headed arrow represents regression.

SEM was used to examine the relationships among the unobservable “latent” variables used in the model [36]. For this type of analysis, a sample size of at least 120 was needed for adequate power [37]. The first stage of the process was the creation of the latent variables and hypothesising the model, followed by confirmatory factor analysis (CFA), which was used to test the relationship between the latent variables of socioeconomic status, diet quality, cardiovascular risk and their respective indications. Once this was conducted, the model was fit using Lavaan version 0.6–5 [38] in R language version 3.6.1 [35]. Due to the presence of ordinal data, the diagonally weighted least squares (DWLS) method was used to produce more accurate parameter estimates. The two loadings for cardiovascular disease risk were fixed to be equal, to allow for free estimation. The term “factor loadings” relates to the standardised coefficients between observed variables and the latent variables [37].

SEM parameters were estimated, and a general fit of the model was assessed. To evaluate the fit of the model, the chi-square statistic value together with the degrees of freedom are reported. However, because chi-square is affected by sample size, four other indices were considered. Ideal goodness of fit was indicated by the comparative fit index (CFI) > 0.9, Tucker–Lewis index (TLI) > 0.9, standardised root mean residual (SRMR) < 0.08 and root mean square error of approximation (RMSEA) < 0.08 [37].

Based on the literature, we hypothesised associations between SES and dyslipidaemia and SES and diet quality. We also expected a causal relationship between diet quality and dyslipidaemia.

## 3. Results

The sample was composed of 50.2% (*n* = 119) girls and 49.8% (*n* = 118) boys, and the mean age was 12.13 (SD = 3.83) years. In terms of ethnicity, the sample was composed of Black children (100%), aged 6–18 years. Most children came from single-headed households (62.7%). Most caregivers had either a primary (43.0%) or secondary education (27.8%), while 17.7% had no formal education, and 3.4% were educated up to tertiary level.

In terms of the children’s dietary intake, the results in Table 1 shows an inadequate per capita intake of 56 grams/day of fruit and vegetable intakes, which does not meet the recommendation of 400 g/day [39]. The mean dietary diversity score was 7.932 ± 1.17. The total fat intake of 48.11 ± 33.22 g/day was equivalent to 25.4% total energy (TE), which is within the 25–35% range [40]. However, added sugar intake was 36.74 ± 31.52 g/day, which is above the recommended range of 12–25 g/day [38]. The total serum cholesterol was 127.02 ± 26.52 mg/dL, and serum LDL-cholesterol was 68.12 ± 24.61 mg/dL, and both were within the recommended range [41].

**Figure 2:**
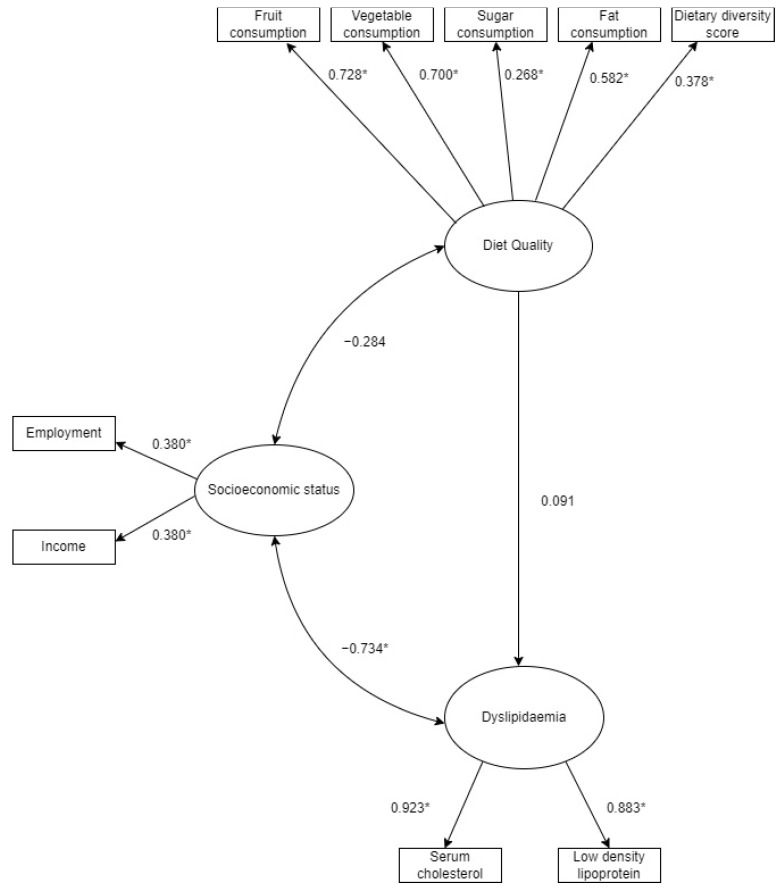
Structural equation model of the relationship between diet quality, socioeconomic status and dyslipidaemia in children. * Statistical significance = *p* < 0.05.

The factor loadings and standardised coefficients, as observed in the structural equation model in Figure 2 and tabulated in Table 2, provide an indication of how well the indicators explained or contributed towards their respective latent variable. As expected, the indicators for diet quality all showed significant factor loadings, with standardised coefficients ranging from 0.268 to 0.728 (*p* < 0.001). The indicators for SES were also statistically significant, and income and employment both had standardised coefficients of 0.380 (*p* = 0.026).

In terms of the relationships among the latent variables, a statistically significant negative covariance was found between SES (based on the indicators employment and income) and dyslipidaemia (based on TC and LDL-C) (−0.734, *p* = 0.029). There was no significant relationship between SES and diet quality or dyslipidaemia and diet quality (fruit and vegetables, added sugar, dietary diversity scores and fat consumption). The results of the structural equation model that explored the relationships between socioeconomic status, diet quality and dyslipidaemia, are highlighted in Table 3, and the goodness of fit of the model is summarised in Table 4.

### Goodness-of-Fit of the Model

The data from all 237 participants were read into lavaan package, but only 178 observations were utilised to estimate the model, due to the fact of missing data, using the DWLS estimator mentioned previously. The chi-square statistic for the model was 53.740 with 32 degrees of freedom (*p* = 0.009). In terms of goodness of fit indices, RMSEA was 0.062 (90% CI: 0.031–0.090) and SRMR was 0.065, both suggesting an acceptable fit. CFI was 0.903 indicating a good fit, but TLI was 0.891 which suggested a mediocre fit. Overall, when considering all indices, the model performed relatively well.

## 4. Discussion

This study set out to explore the relationship between SES, diet quality and CVD risk in children aged six to nineteen years. The results in Table 1 reveal low intakes of fruit and vegetables among the children. Total energy from fat was within the 25–35% range [39], but added sugar intake was high (>10% TE) [40]. Several studies have shown an association between low-income households and high sugar consumption [18,19,20]. At face value, the mean DDS (7.932 SD ± 1.17) and fruit and vegetable intake based on FGDS appeared high, but upon further analysis of the data, it was observed that while different food groups seemed to be consumed, the actual quantities consumed were very low, for instance, on average only 56 g/day of fruit and vegetables were consumed daily, which is just over 25% of the recommended intake [41]. Several studies have shown similar trends with regards to low fruit and vegetable consumption in low-income households both in developed and developing countries [13,14,15,16,17].

Previous research has shown that DDS [34], vegetable intake, fruit intake, fat intake and sugar intake can be used to explain diet quality [7,30]. In this study, these variables were combined to create a composite measure of diet quality and were statistically significant, suggesting that they were good measures of diet quality. The two indicators for dyslipidaemia in this study were high serum cholesterol and LDL-cholesterol, which are well-known risk factors for CVD [24]. As expected, in the model, both total serum cholesterol and LDL-cholesterol had high loadings for dyslipidaemia (*p* < 0.001). Studies have shown the presence of dyslipidaemias in children in countries such as Iran [11], Finland and the USA [22,23,25]. With the rising prevalence of CVD among South Africans [4], it is important for dyslipidaemia in SA children to be identified and addressed early.

Based on our hypothesised model, we expected to find an effect of the latent variable diet quality on dyslipidaemia, as some studies in adults [42,43] and children [44] have observed. However, in our measured model, no significant relationship was observed between diet and dyslipidaemia. Using SEM allowed us to evaluate the relationships based on the overall latent variable of diet quality rather than focusing on one nutrient/food at a time. This provided an alternative representation of the way diet impacts dyslipidaemia in an individual by looking at the combined effect of foods consumed, instead of focusing on individual nutrients/foods one at a time. Thus, although some studies have identified relationships between certain foods and cardiovascular disease, this was not observed in this study when utilising diet quality as a latent variable.

The major finding in this study was a negative association between SES and CVD (−0.734, *p* = 0.029). The influence of SES on dyslipidaemia observed is consistent with findings from studies conducted in Colombian children, Ecuadorian adolescents, as well as adults in both developing and developed countries [7,8,9,10]. However, the results in these studies varied depending on which indicator was used for SES [9] or did not allow for the discernment of the “heterogenous contribution” of different SES measures [10]. In the current study, the use of a latent variable of SES gave us the benefit of not relying on only one SES indicator.

The main strength of this study is that a model was developed to explain the pathways that link SES, diet quality of children and dyslipidaemia. The study used SEM which allowed for testing multiple relationships simultaneously. Additionally, this study provided insight into an understudied area and addressed a gap in the literature. The major finding of this study was that among low SES communities in the Eastern Cape, lower SES was associated with dyslipidaemia. Limitations were also present and can be addressed in future studies. The model was based on data from 178 observations using the diagonally weighted least squares (DWLS) method due to the fact of missing data and presence of ordinal data. While a sample size of 120 is adequate for structural equation modelling [37], a larger sample size could have allowed for sub-analysis based on maturation stage, thus resulting in more age-specific insights (e.g., adolescents). Second, this study was cross-sectional and, as such, we could not determine the temporal effect of exposure and outcome variables. Instead, we were left to assume that SES influenced dyslipidaemia in children, in that order. Since we were limited to observational data, we cannot make any conclusive statements about causation, a challenge common in many epidemiological studies.

## 5. Conclusions

This study successfully modelled the relationship between diet quality, dyslipidaemia and socioeconomic status in students from low-income households in the Eastern Cape. Prior to this study, there were no known studies that investigated the pathways connecting latent variables for diet quality, SES and dyslipidaemia in SA children. Utilising SEM as an analysis strategy may hold potential for new insights to be obtained from nutrition-related data. The results of this study showed a strong inverse association between SES and dyslipidaemia. This population had high levels of poverty, and addressing underlying poverty may be a useful strategy for reducing dyslipidaemia among children of poor SES in the Eastern Cape province and similar populations throughout South Africa. Our findings also highlight the need for more large-scale longitudinal studies that include children from various SES. This would help to examine whether there is a causal relationship between SES and dyslipidaemia and would explore cultural and lifestyle factors that might play a role in differentiating dyslipidaemia in children across various SES groups in SA and other low- and middle-income countries throughout Africa.

## Figures and Tables

**Table 1:** Descriptive statistics for observed variables.

Variable	Mean	Standard Deviation	Reference Values	Classification
Fruit FGDS ^a^	2.257 (32 g/day)	±1.79	400 g/day of fruit and vegetables [39]	Low
Vegetable FGDS ^a^	3.046 (24 g/day)	±1.98		Low
Added Sugar (grams)	36.74	±31.52	12–25 g/day [40]	High
Total Fat (g) and Total Energy (TE)	48.11 g (25.4% TE)	±33.22	25–35% TE depending on age [40]	Moderate
Dietary Diversity Score (DDS)	7.932	±1.17	7–9 food groups [34]	High
Total Serum Cholesterol (mg/dL)	127.02	±26.52	<170 mg/dL [41]	Good
Serum LDL-Cholesterol (mg/dL) ^b^	68.12	±24.61	<100 mg/dL [41]	Good

^a^ Food group diversity score (FGDS); ^b^ low-density lipoprotein (LDL).

**Table 2:** Relationship between latent variables and their indicators.

Latent Variable	Indicator	Factor Loading	Significance (*p*-Value)
SES	Employment	0.380	0.026 *
SES	Income	0.380	0.026 *
Diet Quality	Fruit FGDS ^a^	0.728	<0.001 *
Diet Quality	Vegetable FGDS ^a^	0.700	<0.001 *
Diet Quality	Added Sugar	0.268	<0.001 *
Diet Quality	Dietary Diversity Score	0.582	<0.001 *
Diet Quality	Total Fat Intake	0.373	<0.001 *
Dyslipidaemia	Total Serum Cholesterol	0.923	<0.001 *
Dyslipidaemia	Serum LDL-Cholesterol ^b^	0.883	<0.001 *

* Statistical significance = *p* < 0.05. ^a^ Food group diversity score (FGDS); ^b^ low-density lipoprotein (LDL).

**Table 3:** Structural equation model of the relationships between socioeconomic status, diet quality and dyslipidaemia.

Pathway	Association	Significance (*p*-Value)
^a^ Socioeconomic status and Dyslipidaemia	−0.734	0.029 *
^a^ Socioeconomic status and diet quality	−0.284	0.208
^b^ Dyslipidaemia and diet quality	0.091	0.159

* Statistical significance = *p* < 0.05. ^a^ Covariance; ^b^ regression coefficient.

**Table 4:** Model Fit.

Model	χ^2^	df	RMSEA	RMSEA 90% CI	SRMR	CFI	TLI
SEM	53.740 (*p* = 0.009)	32	0.062	0.031–0.090	0.065	0.903	0.891
			Acceptable		Acceptable	Good	Mediocre/OK

## Data Availability

The data that support the findings of this study are available from the corresponding author, [GM], on request.
